# A randomized, double-blind, positive-controlled, prospective, dose-response clinical study to evaluate the efficacy and tolerability of an aqueous extract of *Terminalia bellerica* in lowering uric acid and creatinine levels in chronic kidney disease subjects with hyperuricemia

**DOI:** 10.1186/s12906-020-03071-7

**Published:** 2020-09-15

**Authors:** Usharani Pingali, Chandrasekhar Nutalapati, Niranjan Koilagundla, Gangadhar Taduri

**Affiliations:** Department of Pharmacology and Therapeutics, Nizam’s Institute of Medical Sciences, Hyderabad, Telangana India

**Keywords:** Terminalia bellerica, Serum creatinine, Uric acid, eGFR

## Abstract

**Background:**

Hyperuricemia is an independent risk factor in chronic kidney disease (CKD). Allopurinol and febuxostat are prescription medicines used to treat hyperuricemia but suffer side-effects. Earlier clinical study has shown that an aqueous extract of *Terminalia bellerica* (TBE), significantly reduced uric acid levels with no serious adverse effects in hyperuricemic subjects. The objective of this study is to determine the efficacy and tolerability of TB in reducing uric acid and creatinine levels in CKD subjects.

**Methods:**

59-subjects were randomized to three groups-40 mg-once-daily febuxostat, 500 mg-twice-daily and 1000 mg-twice-daily of TBE. Serum uric acid, creatinine levels and estimated-glometular-filtration-rate were measured at baseline, 4, 8, 12, 16, 20, 24-weeks. Biomarkers of oxidative-stress, endothelial function, systemic inflammation, and platelet-aggregation were evaluated at baseline, 4, 8, 12, 24-weeks. Adverse drug reactions were recorded. Statistical analysis evaluated using GraphPadPrism4.

**Results:**

55-subjects completed 24-week study. Starting at 4-weeks, all treatment groups showed a significant decrease in serum uric acid levels from baseline (*p* ≤ 0.0001). At 24-weeks, febuxostat, *T.bellerica* 500 mg-twice-daily, and *T.bellerica* 1000 mg-twice-daily doses decreased mean-percentage serum uric acid by 63.70 ± 4.62, 19.84 ± 6.43 and 33.88% ± 4.95% respectively (*p* ≤ 0.0001). Significant decrease in serum creatinine with all the groups starting at 16-weeks was seen (*p* ≤ 0.005-p ≤ 0.0001). At 24-weeks, the mean-percentage change in creatinine levels was 23.71 ± 12.50, 11.70 ± 9.0, and 24.42 ± 8.14, respectively with febuxostat, *T.bellerica* 500 mg-twice-daily and *T.bellerica* 1000 mg-twice-daily. Statistically significant (*p* ≤ 0.05) increase in estimated glomerular filtration rate-(eGFR) was seen at 20 (*p* ≤ 0.05) and 24-weeks (*p* ≤ 0.01) for both febuxostat vs *T.bellerica* 500 mg-twice-daily and *T.bellerica* 1000 mg-twice-daily vs *T.bellerica* 500 mg-twice-daily. There was no statistically significant difference between febuxostat and *T.bellerica* 1000 mg-twice-daily, with an increase of eGFR of 41.38 and 40.39 ml/min/1.73m^2^ respectively, with the inference that *T.bellerica* at 1000 mg-twice-daily dose is as good as febuxostat 40 mg-once-daily. Positive improvements were made by all the groups in endothelial function and the related biomarkers and high-sensitivity C-reactive protein. None of the products showed effect on platelet aggregation.

**Conclusion:**

In this 24-week study Febuxostat 40 mg, *T. bellerica* 500 mg-twice-daily and 1000 mg-twice-daily, significantly decreased the serum uric acid and creatinine levels, increased eGFR in CKD subjects. *T. bellerica* 500 mg-twice-daily and 1000 mg-twice-daily were one-third and more than half as effective at 24-weeks, respectively. *T. bellerica* extract may be considered a natural alternative for reducing serum uric acid levels.

**Trial registration:**

This study was registered with the Clinical Trials Registry – India (CTRI) with the registration number: CTRI/2019/11/022093 [Registered on: 21/11/2019] Trial Registered Retrospectively.

## Background

Chronic kidney disease (CKD) has emerged as a global health problem of epidemic proportions [1]. It may not manifest any pathology but in few may have a progressive and irreversible process requiring renal replacement therapy [2–4]. End-stage renal disease affects the quality of life and may increase the mortality rate [5]. Evidence suggests a high risk of association of hyperuricemia with hypertension, metabolic syndrome, coronary artery disease, cerebrovascular disease, pre-eclampsia, and kidney disease. Decreased renal clearance in subjects with kidney disease frequently leads to hyperuricemia [6]. Hyperuricemia is supposed to be an independent risk factor for CKD [7]. Experimental evidence suggests that uric acid itself may harm subjects with CKD by contributing to increased inflammation and CKD progression [8]. Animal studies have shown that decreasing uric acid levels may slow the progression of CKD [9]. Interventional studies suggest that decreasing uric acid levels in hyperuricemic subjects with CKD is safe and might slow CKD progression. The commonly preferred agents in both overproducers and under-secretors of uric acid include xanthine oxidase (an enzyme involved in the synthesis of uric acid) inhibitors such as allopurinol and febuxostat [10]. In our previous study, we have demonstrated that Terminalia bellerica (TB) significantly decreased serum uric acid levels with no serious adverse effects, thus showing its xanthine oxidase inhibiting property and potential for the treatment of hyperuricemia [11]. Considering this, we carried out the present study to evaluate the effect of TB when compared to febuxostat in preventing the progression of CKD in subjects with hyperuricemia.

## Methods

A total of 59 subjects enrolled in this study conducted at the Department of Clinical Pharmacology and Therapeutics, Nizam’s Institute of Medical Sciences, Hyderabad, India. It was a 24-weeks prospective, randomized, double-blind, placebo-controlled, parallel-group study. Institutional Ethics Committee approved the study. Subjects had willingly given their written informed consent before taking part in the study.

Inclusion criteria included male and female patients aged between 18 and 69 years with serum uric acid level between ≥6.0 mg/dL and ≤ 12.0 mg/dL, not consuming any kind of hypouricemic drugs or stopped taking all uric acid-lowering therapy for at least 2 months before the participation in the study, serum creatinine ≥1.5 mg/dl to ≤3.0 mg/dl and estimated glomerular filtration rate (eGFR) of 30–89 ml/min/1.73 m2 as estimated by modification of diet in renal disease (MDRD) formula, ability to agree with the conditions and requirements of the study and to give written informed consent.

Exclusion criteria included all subjects presenting with gout flare at screening or baseline visit, on aspirin therapy, other nonsteroidal anti-inflammatory drugs (NSAIDs), diuretics, steroids, immunosuppressant drugs, medications with known urate-lowering effects, on an alternative system of medicine, history of any medical conditions like nephrolithiasis, renal transplantation, known liver, thyroid or infectious diseases, uncontrolled hypertension or diabetes, severe hepatic impairment, known or suspected secondary hyperuricemia (e.g. due to myeloproliferative disorder, or organ transplant), pregnant or lactating women, history of chronic alcohol consumption, drug abuse, and history of hypersensitivity to any class of drugs. Subjects complying with the inclusion criteria were only included in the study. After screening, all the eligible subjects were randomized by computer-generated block randomization to either of the three treatment groups in a double-blinded fashion: Group A - Febuxostat 40 mg 1 tablet (encapsulated in a similar capsule to that of TB capsules) + 1 identical placebo capsule taken orally in the morning after food, and 2 identical placebo capsules in the evening after food (40 mg/day dose of febuxostat). Group B – TB 500 mg 1 capsule + 1 identical placebo capsule taken orally in the morning, and 1 capsule of TB 500 mg + 1 identical placebo capsule taken in the evening after food orally (a total dose of 1000 mg/day of TB). Group C – TB 500 mg 2 capsules taken orally in the morning after food, and 2 capsules of TB 500 mg taken in the evening after food orally (a total dose of 2000 mg/day of TB). The bottles containing the test products which were sequentially numbered was dispensed by the pharmacist as per the randomly allocated sequence to the subjects. The principal investigator and the subjects were blinded. The allocations were unblinded to tabulate the data and perform the statistical analyses at the end of the study period.

After obtaining informed consent, the subjects were screened and assessed for the inclusion/exclusion criteria (Visit 1). At visit two (baseline/randomization) after general examination, checking for vital signs and determining routine laboratory investigations, serum creatinine, biomarkers of oxidative stress and serum uric acid, the subjects fulfilling the inclusion criteria were randomized into either of the three study groups as per prior randomization schedule and were dispensed the drugs. The subjects were followed-up at 4, 8, 12, 16, 20, and 24 weeks of the study period. Pill count method was employed to check compliance at each visit. At baseline and at each visit, they were evaluated for efficacy and safety. Serum creatinine, estimated glomerular filtration rate, and serum uric acid levels were measured at baseline and at 4, 8, 12, 16, 20, and 24 weeks. Biomarkers of oxidative stress, reflection index using salbutamol challenge test for determining endothelial function, high sensitivity C-reactive protein (hsCRP), and platelet aggregation test were done at baseline, 4, 12, and 24 weeks of therapy. Safety laboratory investigations for hematological, hepatic, and renal biochemical parameters were done at baseline, at the end of 24 weeks, and, also as and when requiredm (in case of any adverse drug reaction). Subjects were enquired for the presence of any adverse drug reactions and the same was recorded in the case report form. At each visit subjects were dispensed the respective treatments as per prior randomization schedule.

Serum uric acid was done using enzymatic colorimetric test. Serum creatinine was estimated by a kinetic colorimetric assay based on the Jaffe method. The instrument used was Roche Cobas C501 autoanalyzer. Modification of Diet in Renal Disease Study equation was used for estimating glomerular filtration rate.

Endothelial function was evaluated by the salbutamol challenge test using the digital volume plethysmography (DPG) as reported in detail by Chowienczyk et al., Naidu et al., and Millasseau et al. [12–14]. Malondialdehyde in serum was estimated (thiobarbituric acid reactive substance test) spectrophotometrically [15]. Nitric oxide (NO) was estimated using colorimetric detection with Griess reagents [16, 17], whereas Ellman’s method was used to estimate GSH levels [18]. Platelet aggregation test was done with 10μM/mL adenosine diphosphate employing a platelet aggregometry (Chrono-log light transmittance aggregometry) [19].

### Primary and secondary efficacy parameters

The primary efficacy parameter was change in serum uric acid levels at 24 weeks from baseline, change in serum creatinine levels at 24 weeks from baseline, change in estimated glomerular filtration rate at 24 weeks from baseline. Secondary efficacy parameter included a change in reflection Index (a marker of endothelial dysfunction), change in oxidative stress biomarkers (MDA, NO, GSH), changes in systemic inflammation biomarker (hsCRP), effect on platelet aggregation.

### Sample size calculation

The sample size was calculated on the basis that a sample size of 20 in each of the febuxostat and the two *T. bellerica* groups would achieve a power of 80% to detect a clinically significant difference between the group means of uric acid and serum creatinine levels at the end of 24-weeks treatment.

Study data are expressed as mean ± SD. Paired t-test was used to compare the mean change from baseline to post-treatment (4, 8, 12, 16, 20, and 24 weeks of treatment) within each group and ANOVA for between-group comparisons. Post-hoc analysis between the groups was done using Tukey’s test. A *p*-value ≤0.05 was considered statistically significant. Statistical analysis was performed using the software GraphPad PRISM 4.0.

### Study medication

**Fig. 1:**
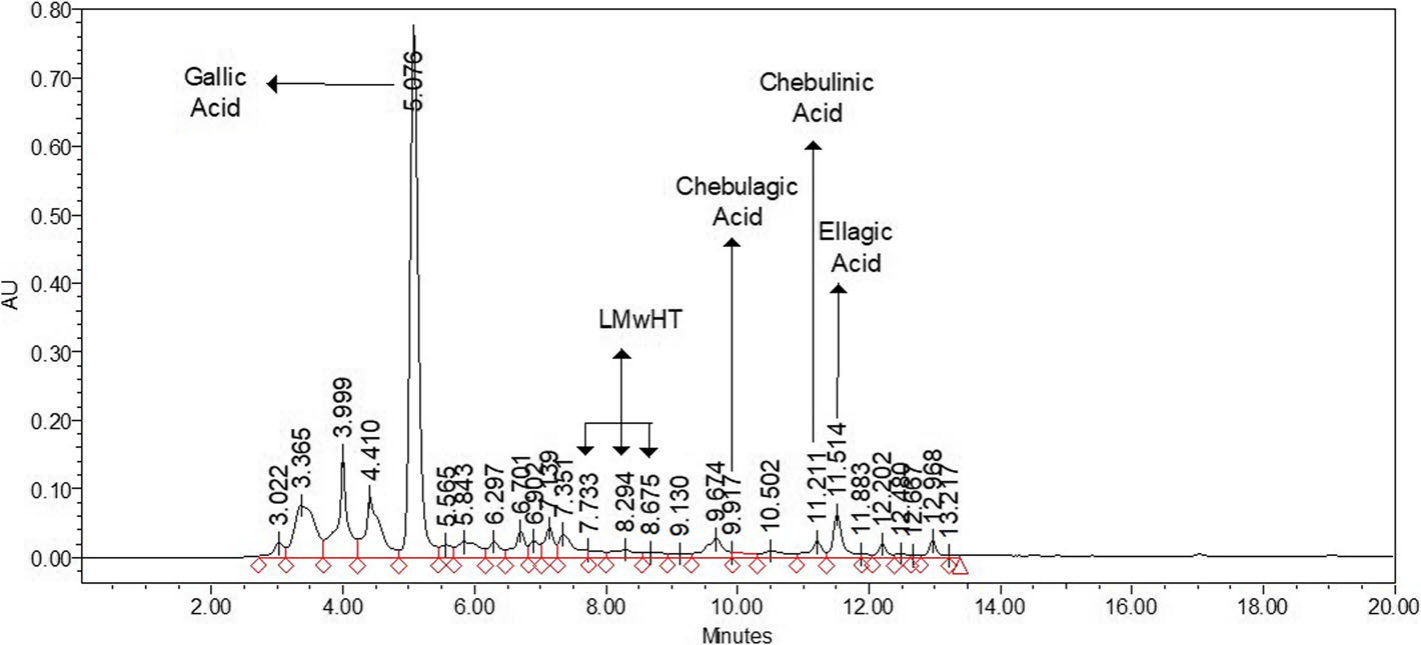
HPLC chromatogram of aqueous extract of Terminalia bellerica

**Fig. 2:**
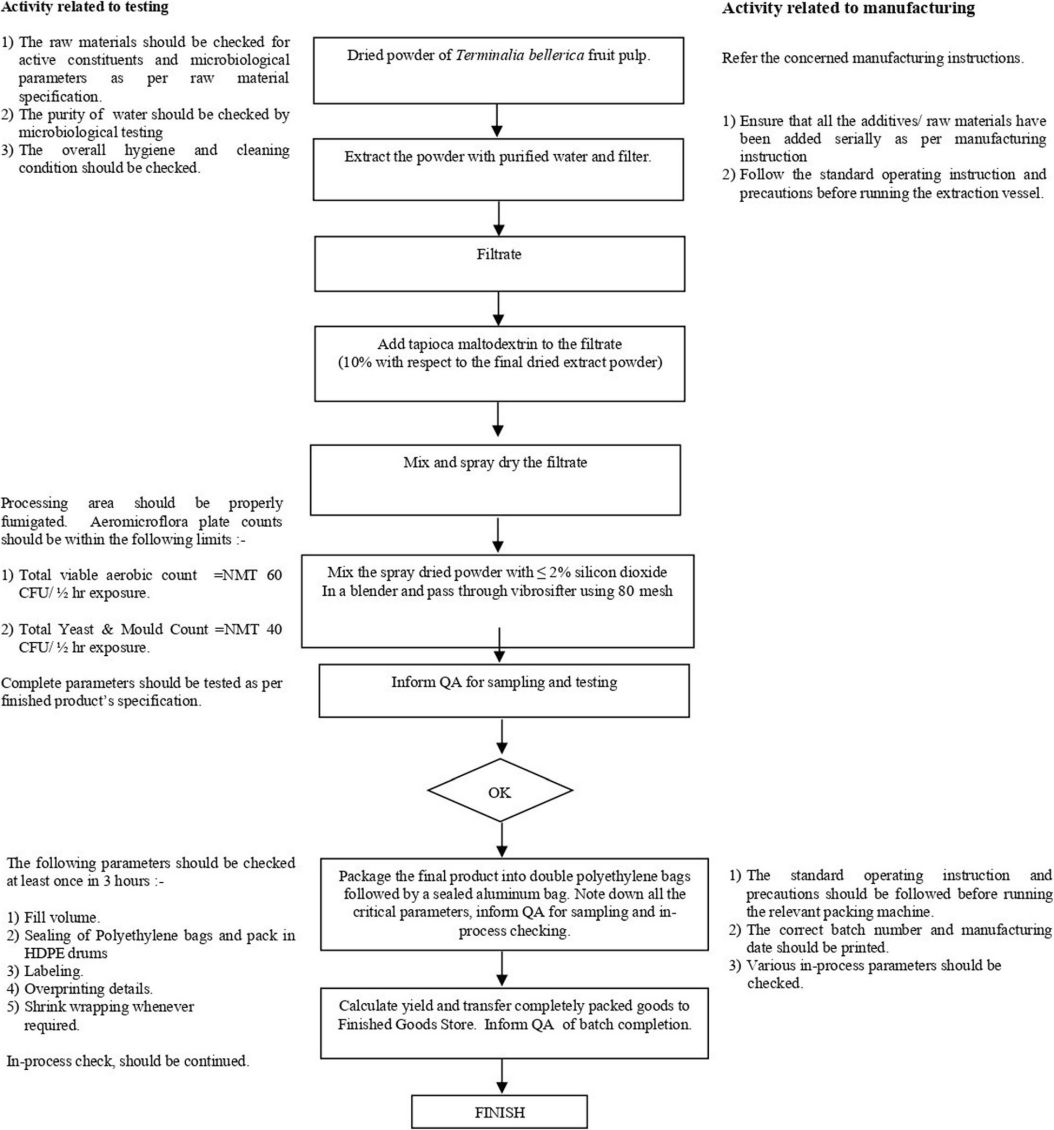
Aquisition of aqueous extract of Terminalia bellerica

Standardized aqueous extract of *Terminalia bellerica* (TB) (Ayuric®) is an aqueous extract of the edible fruits of *T. bellerica* containing not less than 1% of chebulinic acid, not less than 1% of chebulagic acid, and not less than 15% of other low molecular weight hydrolysable tannins, standardized by HPLC, and authenticated by DNA species identification test by Authentechnologies, Richmond, CA. Figures 1 and 2 shows the HPLC chromatogram and acquisition protocol for the aqueous extract of Terminalia bellerica (Ayuric®).

### HPLC conditions

#### Column

The column used for analysis is reverse phase LiChrosob® RP-18, (Particle size 5 μm, 4 × 250 mm) column, Merck KGaA; Germany, with a reverse phase guard column. Temperature: ambient. Eluent: [A]: 1% acetic acid in water, [B]: acetic acid in acetonitrile. Flow rate: 0.7 ml/min. Run time: 20 min.

#### Gradient programme

Table 1

**Table 1:** Gradient programme

Time (min)	Solvent A (%)	Solvent B (%)
0	90	10
15	50	50
16	90	10
20	90	10

#### Detection

UV 270 nm. Injection: 20 μl. Equipment: Waters HPLC 2695 with PDA Detector (Waters™ 2996, Photodiode Array Detector), evaluation with Empower software. Reagents: Pdt. No. UN-1648 Acetonitrile (Merck), Pdt. 93,956 Water for HPLC (Merck). Evaluation: Method with external standard and evaluation of area of peaks using respective calibration equation.

Placebo capsules contains microcrystalline cellulose, croscarmellose sodium, silicon dioxide (fumed), and magnesium stearate. All the study medications were provided by Natreon.

## Results

A total of 68 eligible subjects were screened, 59 were enrolled, and 55 completed the 24 weeks of treatment. Nine subjects were screen failures, while four subjects dropped out of the study before the first follow up. A total of 19 subjects in the febuxostat 40 mg group, 18 subjects in TB 500 mg twice daily group, and 18 subjects in TB 1000 mg twice daily group completed the 24 weeks of study treatment.

As shown in Table 2, there were no significant differences between treatment groups in baseline characteristics including age, weight & body mass index (BMI), indicating a homogenous population.

**Table 2:** Demographic data

Parameter	Febuxostat 40 mg OD (A)	TB 500 mg BID (B)	TB 1000 mg BID (C)
No. of subjects	19	18	18
Gender (M/F)	14 M / 5F	13 M / 5F	14 M / 4F
Age (Years)	50.95 ± 9.82	53.17 ± 8.91	50.78 ± 8.79
BMI (Kg/m^2^)	26.78 ± 1.29	25.52 ± 0.87	25.63 ± 1.61

*OD* once daily, *BID* twice daily

As depicted in Tables 3 and 4, there was a significant decrease in serum creatinine levels with all the groups starting at 16 weeks and continuing until the end of the study (*p* ≤ 0.005 - *p* ≤ 0.0001). At 24 weeks, the mean percentage change in creatinine levels was 23.71 ± 12.50, 11.70 ± 9.0, and 24.42 ± 8.14 mg/dL respectively with febuxostat, TB 500 mg twice daily and TB 1000 mg twice daily groups. No statistically significant difference was seen in the mean percentage reduction of serum creatinine levels at the end of 4, 8, and 12 weeks between the groups. However, statistically significant (*p* ≤ 0.05) difference was seen at 16 weeks for TB 1000 mg twice daily group (C) vs TB 500 mg twice daily group (B) and at 20 and 24 weeks of treatment for febuxostat 40 mg (A) versus TB 500 mg twice daily (B), and for TB 1000 mg twice daily (C) vs TB 500 mg twice daily (B). There was no statistically significant difference between groups C and A, which means that TB at 1000 mg twice daily dose performed as good as febuxostat at 40 mg once daily dose in reducing serum creatinine levels, with mean serum creatinine levels decreasing by 23.71 and 24.42% respectively in 24 wks.

**Table 3:** Serum creatinine levels, mg/dL

Group	Baseline	4 weeks	Mean%change	8 weeks	Mean%change	12 weeks	Mean%change	16 weeks	Mean%change	20 weeks	Mean%change	24 weeks	Mean%change
A	2.00 ± 0.27	2.01 ± 0.31	0.57 ± 8.50	1.93 ± 0.29	−3.42 ± 9.69	1.81 ± 0.35	−9.53 ± 12.96	1.74 ± 0.36	− 13.38 ± 13.34	1.61 ± 0.29	−19.36 ± 10.81	1.52 ± 0.30	− 23.71 ± 12.50
B	1.86 ± 0.32	1.84 ± 0.32	−1.22 ± 3.21	1.88 ± 0.31	1.03 ± 4.48	1.78 ± 0.31	− 4.39 ± 5.92	1.74 ± 0.32	− 6.71 ± 4.57	1.68 ± 0.30	−9.52 ± 7.06	1.64 ± 0.29	− 11.70 ± 9.00
C	2.06 ± 0.26	2.01 ± 0.28	−2.47 ± 5.55	1.97 ± 0.31	− 4.52 ± 7.23	1.83 ± 0.30	− 11.13 ± 9.81	1.74 ± 0.30	− 15.44 ± 10.23	1.64 ± 0.27	− 20.24 ± 8.81	1.56 ± 0.24	− 24.42 ± 8.14

**Table 4:** *p* values for change in serum creatinine levels within the groups (from baseline to the end of the treatment period) and between the groups

Group	4 weeks	8 weeks	12 weeks	16 weeks	20 weeks	24 weeks
A	ns	ns	*p* ≤ 0.005	*p* ≤ 0.005	*p* ≤ 0.0001	*p* ≤ 0.0001
B	ns	ns	*p* ≤ 0.005	*p* ≤ 0.0001	*p* ≤ 0.0001	*p* ≤ 0.005
C	ns	*p* ≤ 0.05	*p* ≤ 0.0001	*p* ≤ 0.0001	*p* ≤ 0.0001	*p* ≤ 0.0001
A vs B	ns	ns	ns	ns	*p* ≤ 0.005	*p* ≤ 0.005
A vs C	ns	ns	ns	ns	ns	ns
C vs B	ns	ns	ns	*p* ≤ 0.05	*p* ≤ 0.005	*p* ≤ 0.005

Tables 5 and 6 shows that no statistical significance was seen in the mean percentage increase in eGFR at the end of 4, 8, 12, 16 weeks between the groups. However, a statistically significant (*p* ≤ 0.05) increase was seen at 20 (*p* ≤ 0.05) and 24 weeks (*p* ≤ 0.01) for both febuxostat vs TB 500 mg twice daily, and TB 1000 mg twice daily vs TB 500 mg twice daily groups. Again, there was no statistically significant difference between febuxostat and TB 1000 mg twice daily groups, with an increase of eGFR of 41.38 and 40.39 ml/min/1.73m^2^ respectively, with the inference that TB at 1000 mg twice daily dose is as good as febuxostat 40 mg once daily in its effect on eGFR.

**Table 5:** eGFR, mil/min/1.73m^2^

Group	Baseline	4 weeks	Mean%change	8 weeks	Mean%change	12 weeks	Mean%change	16 weeks	Mean%change	20 weeks	Mean%change	24 weeks	Mean%change
A	35.58 ± 4.98	35.76 ± 6.84	0.10 ± 8.80	37.46 ± 6.75	5.33 ± 12.13	41.07 ± 9.75	15.29 ± 21.41	43.26 ± 10.18	21.81 ± 24.92	46.45 ± 8.92	31.11 ± 22.00	50.01 ± 10.65	41.38 ± 28.79
B	39.13 ± 6.57	39.71 ± 6.66	1.56 ± 4.21	38.76 ± 6.86	−0.96 ± 4.78	41.42 ± 7.90	5.77 ± 7.28	42.63 ± 8.36	8.65 ± 6.11	44.31 ± 9.01	13.06 ± 10.28	45.96 ± 11.14	16.96 ± 14.87
C	34.78 ± 5.34	35.98 ± 6.41	3.35 ± 7.39	37.20 ± 8.79	6.25 ± 10.00	40.92 ± 11.96	16.52 ± 17.46	43.40 ± 12.29	23.91 ± 21.37	46.22 ± 12.16	32.28 ± 22.47	48.93 ± 11.46	40.39 ± 20.98

**Table 6:** *P* values for change in eGFR levels within the groups (from baseline to the end of the treatment period) and between the groups

Group	4 weeks	8 weeks	12 weeks	16 weeks	20 weeks	24 weeks
A	ns	ns	*p* ≤ 0.005	*p* ≤ 0.005	*p* ≤ 0.0001	*p* ≤ 0.0001
B	ns	ns	p ≤ 0.005	*p* ≤ 0.0001	*p* ≤ 0.0001	*p* ≤ 0.005
C	ns	*p* ≤ 0.05	*p* ≤ 0.01	*p* ≤ 0.005	*p* ≤ 0.0001	*p* ≤ 0.0001
A vs B	ns	ns	ns	ns	*p* ≤ 0.05	*p* ≤ 0.01
A vs C	ns	ns	ns	ns	ns	ns
C vs B	ns	ns	ns	ns	*p* ≤ 0.05	*p* ≤ 0.01

As seen in Tables 7 and 8, all the three groups decreased serum uric acid levels starting at 4 weeks and continuing until the end of the study (*p* ≤ 0.0001). TB 1000 mg twice daily group peaked at 20 weeks at 34.7%, while the febuxostat group peaked at 24 weeks with a decrease of 63.7%, consistently performing better than both the TB groups (*p* ≤ 0.001). TB 1000 mg twice daily group also performed significantly better (*p* ≤ 0.001) than the 500 mg twice daily group starting at 16 weeks.

**Table 7:** Serum uric acid levels, mg/dL

Group	Baseline	4 weeks	Mean %change	8 weeks	Mean %change	12 weeks	Mean %change	16 weeks	Mean %change	20 weeks	Mean %change	24 weeks	Mean %change
A	9.04 ± 0.64	7.98 ± 0.49	−11.55 ± 4.32	6.63 ± 0.32	−26.37 ± 5.54	4.78 ± 0.60	− 46.80 ± 8.40	3.95 ± 0.59	− 56.16 ± 7.32	3.83 ± 0.52	−57.50 ± 6.16	3.28 ± 0.46	−63.70 ± 4.62
B	8.10 ± 0.67	7.64 ± 0.69	− 5.72 ± 2.15	7.10 ± 0.61	−12.34 ± 2.12	6.95 ± 0.51	−14.07 ± 3.49	6.76 ± 0.37	− 16.25 ± 4.88	6.59 ± 0.27	−18.28 ± 5.47	6.46 ± 0.34	− 19.84 ± 6.43
C	8.54 ± 0.64	8.06 ± 0.66	− 5.70 ± 1.67	7.87 ± 0.70	− 7.87 ± 2.58	6.98 ± 0.70	− 18.32 ± 4.91	6.08 ± 0.55	− 28.67 ± 5.09	5.56 ± 0.41	− 34.70 ± 4.76	5.63 ± 0.37	− 33.88 ± 4.95

**Table 8:** *P* values for change in serum uric acid levels within the groups (from baseline to the end of the treatment period) and between the groups

Group	4 weeks	8 weeks	12 weeks	16 weeks	20 weeks	24 weeks
A	*p* ≤ 0.0001	*p* ≤ 0.0001	*p* ≤ 0.0001	*p* ≤ 0.0001	*p* ≤ 0.0001	*p* ≤ 0.0001
B	*p* ≤ 0.0001	*p* ≤ 0.0001	*p* ≤ 0.0001	*p* ≤ 0.0001	*p* ≤ 0.0001	*p* ≤ 0.0001
C	*p* ≤ 0.0001	*p* ≤ 0.0001	*p* ≤ 0.0001	*p* ≤ 0.0001	*p* ≤ 0.0001	*p* ≤ 0.0001
A vs B	*p* ≤ 0.001	*p* ≤ 0.001	*p* ≤ 0.001	*p* ≤ 0.001	*p* ≤ 0.001	*p* ≤ 0.001
A vs C	*p* ≤ 0.001	*p* ≤ 0.001	*p* ≤ 0.001	*p* ≤ 0.001	*p* ≤ 0.001	*p* ≤ 0.001
C vs B	ns	*p* ≤ 0.01	ns	*p* ≤ 0.001	*p* ≤ 0.001	*p* ≤ 0.001

The endothelium is essential in preserving vascular homeostasis. Apart from its vasodilator properties, a healthy endothelium also prevents atherosclerosis by inhibition of platelet aggregation and adhesion, smooth muscle cell proliferation and leukocyte adhesion. However, disruption in the activity of endothelial cell may be a triggering factor for atherosclerosis and ensuing cardiac events [20]. Reflection Index (RI) is a measure of endothelial function [21]. As seen in Tables 9 and 10, the decrease in RI (%) in the febuxostat group is statistically significant compared to either 500 mg twice daily or the 1000 mg twice daily TB groups. However, even with febuxostat, improvement in endothelial function appears to be marginal at − 6.27%, the definition of endothelial dysfunction being less than 6% decrease in RI.

**Table 9:** Change in RI, %

Group	Baseline	4 weeks	Absolute change	12 weeks	Absolute change	24 weeks	Absolute change
A	−2.39 ± 0.54	−3.33 ± 0.59	−0.93 ± 0.27	−4.71 ± 0.67	−2.32 ± 0.48	−6.27 ± 0.73	−3.88 ± 0.67
B	−3.18 ± 0.63	−3.49 ± 0.64	−0.31 ± 0.15	−3.89 ± 0.57	−0.71 ± 0.23	− 4.35 ± 0.57	−1.17 ± 0.32
C	−2.57 ± 0.74	−3.32 ± 0.67	−0.76 ± 0.45	−3.79 ± 0.63	−1.22 ± 0.44	− 4.82 ± 0.57	− 2.25 ± 0.56

**Table 10:** *P* values for change in RI within the groups (from baseline to the end of the treatment period) and between the groups

Group	4 weeks	12 weeks	24 weeks
A	*p* ≤ 0.0001	*p* ≤ 0.0001	*p* ≤ 0.0001
B	*p* ≤ 0.0001	*p* ≤ 0.0001	*p* ≤ 0.0001
C	*p* ≤ 0.0001	*p* ≤ 0.0001	*p* ≤ 0.0001
A vs B	*p* ≤ 0.001	*p* ≤ 0.001	*p* ≤ 0.001
A vs C	ns	*p* ≤ 0.001	*p* ≤ 0.001
C vs B	*p* ≤ 0.001	*p* ≤ 0.001	*p* ≤ 0.001

Malondialdehyde (MDA) is a lipid peroxidation product, and its levels increase in the body as a result of increased oxidative stress [22]. It is the most frequently used biomarker of oxidative stress in many health problems such as cancer, psychiatry, chronic obstructive pulmonary disease, asthma, or cardiovascular diseases despite its unreliability [23]. As shown in the Tables 11 and 12, by the end of 24 weeks, TB 1000 mg twice daily and 500 mg twice daily dosages decreased MDA levels by 9.6 and 7.2% respectively, while febuxostat decreased it by 17.26%. The decrease in MDA levels by febuxostat was significantly (*p* ≤ 0.001) better than either group of TB.

**Table 11:** MDA, μM/L

Groups	Baseline	4 weeks	Mean % Change	12 weeks	Mean % Change	24 weeks	Mean % Change
A	3.60 ± 0.51	3.43 ± 0.45	−4.72 ± 3.05	3.28 ± 0.44	−8.65 ± 3.80	2.97 ± 0.39	− 17.26 ± 4.78
B	4.04 ± 0.55	3.99 ± 0.52	−0.94 ± 1.57	3.87 ± 0.48	−3.92 ± 1.80	3.74 ± 0.46	− 7.20 ± 2.40
C	3.23 ± 0.47	3.15 ± 0.48	−2.61 ± 1.24	3.05 ± 0.47	−5.62 ± 1.20	2.92 ± 0.40	− 9.60 ± 3.13

**Table 12:** *P* values for change in MDA levels within the groups (from baseline to the end of the treatment period) and between the groups

Statistics	4 weeks	12 weeks	24 weeks
A	*p* ≤ 0.0001	*p* ≤ 0.0001	*p* ≤ 0.0001
B	*p* ≤ 0.05	*p* ≤ 0.0001	*p* ≤ 0.0001
C	*p* ≤ 0.0001	*p* ≤ 0.0001	*p* ≤ 0.0001
A vs B	*p* ≤ 0.001	*p* ≤ 0.001	*p* ≤ 0.001
A vs C	*p* ≤ 0.05	*p* ≤ 0.01	*p* ≤ 0.001
C vs B	ns	ns	ns

Nitric oxide (NO) plays an essential role in maintaining good vascular health by exerting antiplatelet, antithrombotic, and anti-inflammatory activity. A common abnormality found in many vascular diseases is endothelial dysfunction which can be due to impaired NO bioavailability. Insufficiency in NO levels can be related to limited substrate/cofactor availability as well as interactions with reactive oxygen species (ROS) [24]. As evidenced by the data in Tables 13 and 14, the mean percentage increase in NO levels at 24 weeks by TB 1000 mg twice daily group and the febuxostat group was significant at 12.02 and 13.47% with no statistical difference between these two groups. Both these groups were significantly better in increasing NO levels compared to the TB 500 mg twice daily group, which increased it by 9.35%.

**Table 13:** NO, μM/L

Groups	Baseline	4 weeks	Mean % Change	12 weeks	Mean % Change	24 weeks	Mean % Change
A	31.04 ± 2.05	32.17 ± 2.21	3.64 ± 1.85	33.53 ± 2.10	8.08 ± 2.28	35.18 ± 1.83	13.47 ± 3.13
B	29.62 ± 2.19	30.46 ± 2.18	2.86 ± 0.75	31.35 ± 2.06	5.92 ± 1.32	32.36 ± 2.05	9.35 ± 1.60
C	29.69 ± 1.99	30.76 ± 1.88	3.63 ± 1.00	32.10 ± 2.17	8.10 ± 1.65	33.25 ± 2.10	12.02 ± 1.58

**Table 14:** *P* values for change in NO levels within the groups (from baseline to the end of the treatment period) and between the groups

Group	4 weeks	12 weeks	24 weeks
A	*p* ≤ 0.0001	*p* ≤ 0.0001	*p* ≤ 0.0001
B	*p* ≤ 0.0001	*p* ≤ 0.0001	*p* ≤ 0.0001
C	*p* ≤ 0.0001	*p* ≤ 0.0001	*p* ≤ 0.0001
A vs B	ns	*p* ≤ 0.01	*p* ≤ 0.001
A vs C	ns	ns	ns
C vs B	ns	*p* ≤ 0.01	*p* ≤ 0.01

Glutathione (GSH) is an endogenous antioxidant and is known to minimize lipid peroxidation resulting from excessive ROS [25, 26]. As seen from Tables 15 and 16 mean percentage increase in GSH levels at 24 weeks by febuxostat, TB 1000 mg twice daily, and TB 500 mg twice daily groups was significant (*p* ≤ 0.0001) at 7.7, 8.02, and 4.33% respectively. The first two groups, with no statistical difference between them, performed significantly better than the TB 500 mg twice daily group.

**Table 15:** GSH, μM/L

Groups	Baseline	4 weeks	Mean % Change	12 weeks	Mean % Change	24 weeks	Mean % Change
A	541.95 ± 20.88	550.83 ± 20.55	1.64 ± 0.40	567.07 ± 21.60	4.64 ± 0.74	583.69 ± 23.73	7.70 ± 1.14
B	550.30 ± 18.46	556.02 ± 19.58	1.04 ± 0.29	568.96 ± 23.64	3.37 ± 1.25	574.19 ± 23.21	4.33 ± 1.31
C	536.79 ± 15.24	544.34 ± 15.44	1.41 ± 0.23	560.94 ± 14.72	4.51 ± 0.80	579.82 ± 15.86	8.02 ± 1.18

**Table 16:** p values for change in GSH levels within the groups (from baseline to the end of the treatment period) and between the groups

Group	4 weeks	12 weeks	24 weeks
A	*p* ≤ 0.0001	*p* ≤ 0.0001	*p* ≤ 0.0001
B	*p* ≤ 0.0001	*p* ≤ 0.0001	*p* ≤ 0.0001
C	*p* ≤ 0.0001	*p* ≤ 0.0001	*p* ≤ 0.0001
A vs B	*p* ≤ 0.001	*p* ≤ 0.001	*p* ≤ 0.0001
A vs C	ns	ns	ns
C vs B	*p* ≤ 0.01	*p* ≤ 0.01	*p* ≤ 0.0001

One of the reliable biomarkers of chronic systemic inflammation includes C- reactive protein. Impaired levels of high sensitivity C- reactive protein (hs-CRP) may be indicative of metabolic syndrome and its components. It may also be of prognostic importance on the future development of cardiovascular events. hs-CRP levels less than 1, 1 to 3, and greater than 3 mg/L indicate a low, moderate, and high risk for future CHD events and stroke. In the present study, the baseline hsCRP values were more than 6 mg/L, indicating a very high cardiovascular event risk [27]. As seen from Tables 17 and 18 mean percentage decrease in hsCRP levels by all the groups was significant at almost all treatment periods. At 24 weeks, the decrease in hsCRP level by TB 1000 mg twice daily and the febuxostat groups was significant at 13.76 and 19.66% respectively, with no statistical difference between these two groups. Both these groups were significantly better in decreasing hsCRP levels compared to the TB 500 mg twice daily group, which decreased hsCRP by 9.86%. The normal range for platelet aggregation using adenosine diphosphate (10 μM/ml) is between 60 to 90%. To show if the drug affects platelet aggregation, there should be more than 30% change in percent inhibition. In the present study the mean percentage inhibition in Group A, Group B, and Group C at the end of 24 weeks of study was 0.88, 1.57, and 1.34%, respectively, which is minimal indicating that none of the groups affected platelet aggregation.

**Table 17:** hsCRP, mg/L

Groups	Baseline	4 weeks	Mean % Change	12 weeks	Mean % Change	24 weeks	Mean % Change
A	7.37 ± 0.97	7.08 ± 0.95	− 3.96 ± 1.49	6.63 ± 0.97	− 10.14 ± 2.31	5.94 ± 0.95	−19.66 ± 3.46
B	7.80 ± 0.97	7.73 ± 0.97	− 0.86 ± 1.18	7.41 ± 0.92	− 5.05 ± 1.98	7.03 ± 0.92	− 9.86 ± 2.68
C	8.41 ± 1.11	8.11 ± 1.10	− 3.66 ± 1.32	7.72 ± 1.07	−8.30 ± 2.33	7.26 ± 1.04	− 13.76 ± 2.53

**Table 18:** p values for change in hsCRP levels within the groups (from baseline to the end of the treatment period) and between the groups

Group	4 weeks	12 weeks	24 weeks
A	*p* ≤ 0.0001	*p* ≤ 0.0001	*p* ≤ 0.0001
B	*p* ≤ 0.01	*p* ≤ 0.0001	*p* ≤ 0.0001
C	*p* ≤ 0.0001	*p* ≤ 0.0001	*p* ≤ 0.0001
A vs B	*p* ≤ 0.001	*p* ≤ 0.001	*p* ≤ 0.001
A vs C	ns	*p* ≤ 0.05	*p* ≤ 0.001
C vs B	*p* ≤ 0.001	*p* ≤ 0.001	*p* ≤ 0.001

All safety hematological and biochemical parameters were within normal limits in all three treatment groups at the end of the study. A total of 4 subjects in the febuxostat group had reported complaints of nausea and vomiting. Two subjects in TB groups had mild gastrointestinal intolerance. However, no subject in either group discontinued the study due to adverse events.

## Discussion

In the present study done on CKD patients with hyperuricemia, we have shown a significant improvement in serum uric acid levels with all the three treatment groups. Febuxostat had shown the best effect, followed by TB 1000 mg twice daily and TB 500 mg twice daily. Similarly, a significant improvement in the levels of serum creatinine was observed with the treatment groups. The effect was seen from the 8th week onwards with the maximum effect observed at the end of 24 weeks of treatment. Also, a significant improvement in the estimated glomerular filtration rate levels with all the treatment groups was observed starting from 8th week onwards till the end of 24 weeks of study. The maximum effect was observed by febuxostat and TB 1000 mg twice daily.

In a study by Kimura et al. [28], which was done to evaluate the effect of febuxostat on stage 3 CKD patients with asymptomatic hyperuricemia, it was reported that a significant improvement in estimated glomerular filtration rate and serum uric acid levels was seen with febuxostat at the end of the study. Another study by Sircar et al. [29] reported improvement in GFR and serum uric acid levels over a period of 6 months with febuxostat in CKD patients with hyperuricemia. Tsuruta et al. [30] reported significant improvement in serum uric acid and estimated glomerular filtration rate with 40 mg of febuxostat when compared to allopurinol in patients of CKD. Our study also has reported similar results.

A multicenter study to evaluate the association of uric acid, serum creatinine and diuretic use in hypertension patients by Lin et al. [31] reported that elevated uric acid levels were associated with increased serum creatinine levels. A study by Kim Ah Hyun et al. [32] done on subjects with gout reported that serum creatinine decreased in the febuxostat group, which was correlated to significant changes in uric acid level.

In a previous study [11] done at our department, we have reported that *T. bellerica* inhibited uric acid formation, which was indicative of its xanthine oxidase enzyme inhibitory activity. In our present study, it is observed that serum uric acid and serum creatinine levels have significantly improved, which in turn has led to the improvement in estimated glomerular filtration rate with all the treatment groups. We correlate the *T. bellerica’s* property of improving uric acid levels in the present study to its xanthine oxidase inhibitory property, which in turn has improved serum creatinine as well as estimated glomerular filtration rate.

There is evidence suggesting the vital role of endothelial dysfunction in kidney disease. Chronic kidney disease itself may have a contributory role in endothelial dysfunction by escalating inflammatory process and oxidative stress. Preclinical and clinical studies have established the role of oxidative stress in CKD. Elevated uric acid seems to contribute to endothelial dysfunction, inflammatory process and oxidative stress. Considering this aspect, we evaluated the endothelial function and oxidative stress biomarkers in our study [33, 34]..

In a 4-week study by Tsuruta et al. [35] in hemodialysis patients with hyperuricemia, febuxostat significantly improved endothelial function as assessed by flow-mediated dilation and decrease in MDA-LDL levels. Another study by Fahmi et al. [36] reported that febuxostat reversed cisplatin-induced elevation in renal MDA levels and attenuated depletion of renal and antioxidant defense system (as measured by GSH levels and superoxide dismutase activity). Febuxostat also elevated renal NO levels to near normal. They attributed this improvement to the antioxidant effects of febuxostat via inhibition of xanthine oxidase derived reactive oxygen species. Hwang JS et al. [37] in their study to evaluate the effect of febuxostat on endothelial dysfunction in streptozocin-induced diabetic rats reported that febuxostat inhibited xanthine oxidase and promoted nitric oxide release. Malondialdehyde had shown a decreasing trend in their study. They noted that febuxostat improved endothelial function via attenuating oxidative stress by XO inhibition. Shirakura T et al. [38] have reported that Febuxostat an XO inhibitor improves hypertension and endothelial function in spontaneously hypertensive rats as measured by tissue nitrotyrosine levels a marker of nitro-oxidative stress. Nitrotyrosine is formed from the scavenge of NO by ROS, which reacts rapidly with NO to form peroxynitrite, which in turn induces endothelial function. Hence attenuation of NO-scavenge and suppression of peroxynitrite ameliorates endothelial function. Similarly, in our study, we have reported a significant improvement in the RI (a marker of endothelial function), MDA, NO, and GSH levels with all the treatment groups. The improvement in the *T. bellerica* group also was significant. The probable mechanism of action of *T. bellerica* could be akin to those mentioned above.

Sezai et al. [39], in a study done to compare the effect of febuxostat and allopurinol for hyperuricemia in cardiac surgery patients with chronic kidney disease have shown improvement in hs-CRP levels at the end of 6 months. We have also shown a modest improvement in the hs-CRP levels with febuxostat. Improvement was observed with TB group also with TB 1000 mg twice daily showing a better result than TB 500 mg twice daily, thus indicating its potential anti-inflammatory property.

## Conclusion

Standardized aqueous extract of *T. bellerica*, at 500 mg and 1000 mg twice daily doses, significantly decreased the serum uric acid and creatinine levels and increased the estimated glomerular filtration rate in a 24-week clinical study in chronic kidney disease subjects with hyperuricemia. Compared to the positive control, febuxostat at 40 mg dose, the *T. bellerica* 500 mg twice daily group appeared to be one third as effective, while the 1000 mg twice daily group was more than half as effective at 24 weeks. In addition, there were significant improvements in oxidative stress biomarkers as well as the systemic inflammation biomarker with all the groups. *T. bellerica* extract may be considered a natural alternative for reducing serum uric acid levels.

## Supplementary information

**Additional file 1.** Consort flow diagram

## Data Availability

The datasets used or analyzed during the current study are available from the corresponding author on reasonable request.

## Abbreviations

BMI: Body mass index
CKD: Chronic kidney disease
eGFR: Estimated glomerular filtration rate
GSH: Glutathione
hsCRP: High-sensitivity C-reactive protein
HPLC: High-performance liquid chromatography
MDA: Malondialdehyde
MDA-LDL: Malondialdehyde-modified low-density lipoprotein
NO: Nitric oxide
NSAIDs: Nonsteroidal anti-inflammatory drugs
RI: Reflection index
ROS: Reactive oxygen species
SD: Standard deviation
TB: *Terminalia bellerica*
T. bellerica: *Terminalia bellerica*
XO: Xanthine oxidase
