# Supplementary figures and images for: A randomized, double-blind, positive-controlled, prospective, dose-response clinical study to evaluate the efficacy and tolerability of an aqueous extract of Terminalia bellerica in lowering uric acid and creatinine levels in chronic kidney disease subjects with hyperuricemia
Source: BMC Complement Med Ther. 2020 Sep 15;20:281. doi: 10.1186/s12906-020-03071-7 (PMC7493401; doi:10.1186/s12906-020-03071-7)

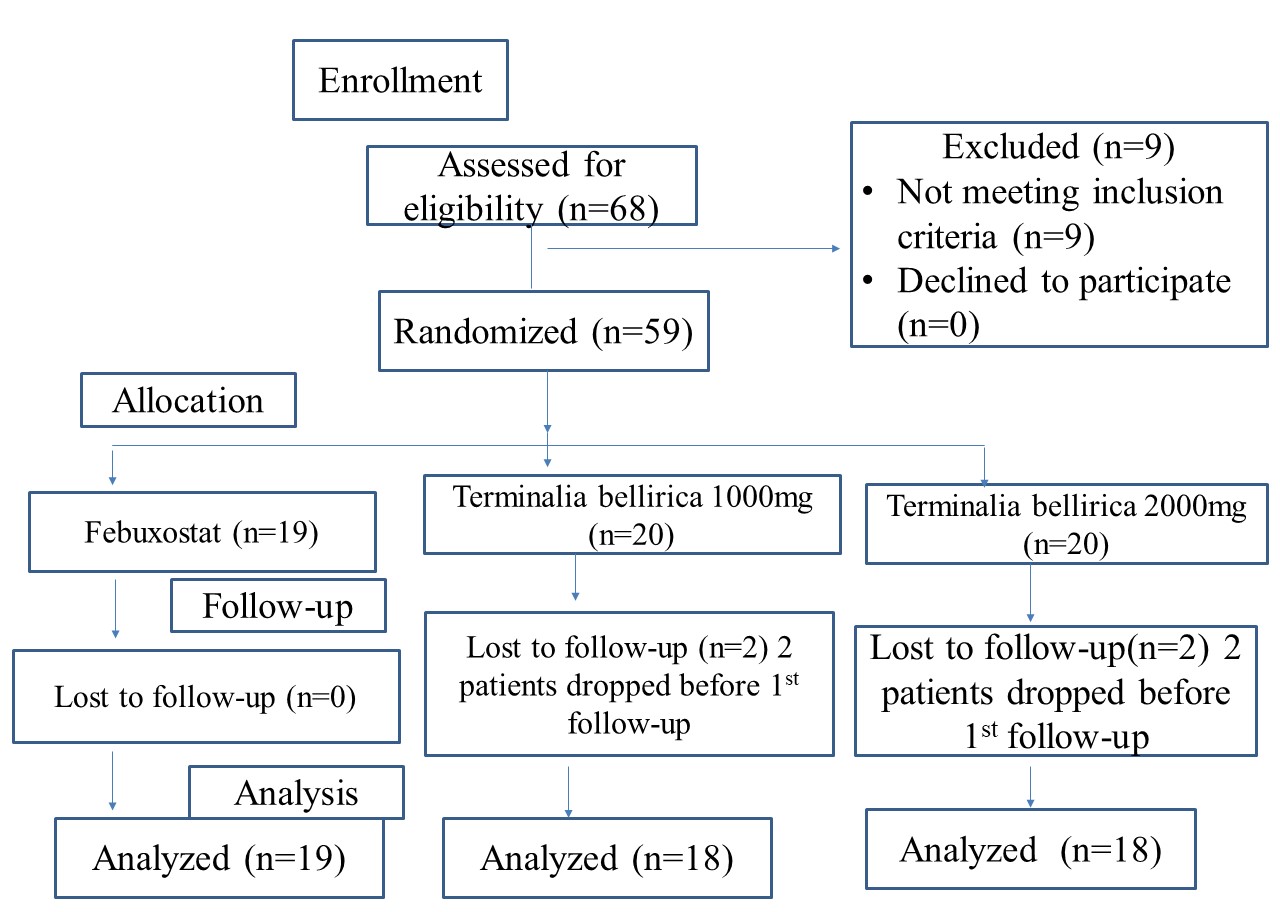

Supplement: Supplementary file 1 — Additional file 1. Consort flow diagram [file 12906_2020_3071_MOESM1_ESM.jpg]
